# Supplementary material for: A Possible Inhibitory Role of Sialic Acid on MUC1 in Peritoneal Dissemination of Clear Cell-Type Ovarian Cancer Cells
Source: Molecules. 2021 Oct 1;26(19):5962. doi: 10.3390/molecules26195962 (PMC8512441; doi:10.3390/molecules26195962)

Supplementary Figures S1-S3

Title: A possible inhibitory role of sialic acid on MUC1 in peritoneal dissemination of clear cell-type ovarian cancer cells

Authors: Yutaka Tamada, Hiroyuki Nomura, Daisuke Aoki, Tatsuro Irimura

Effect of sialidase treatment of three ovarian cancer cell lines on adhesion to peritoneal mesothelial cells *in vitro*.

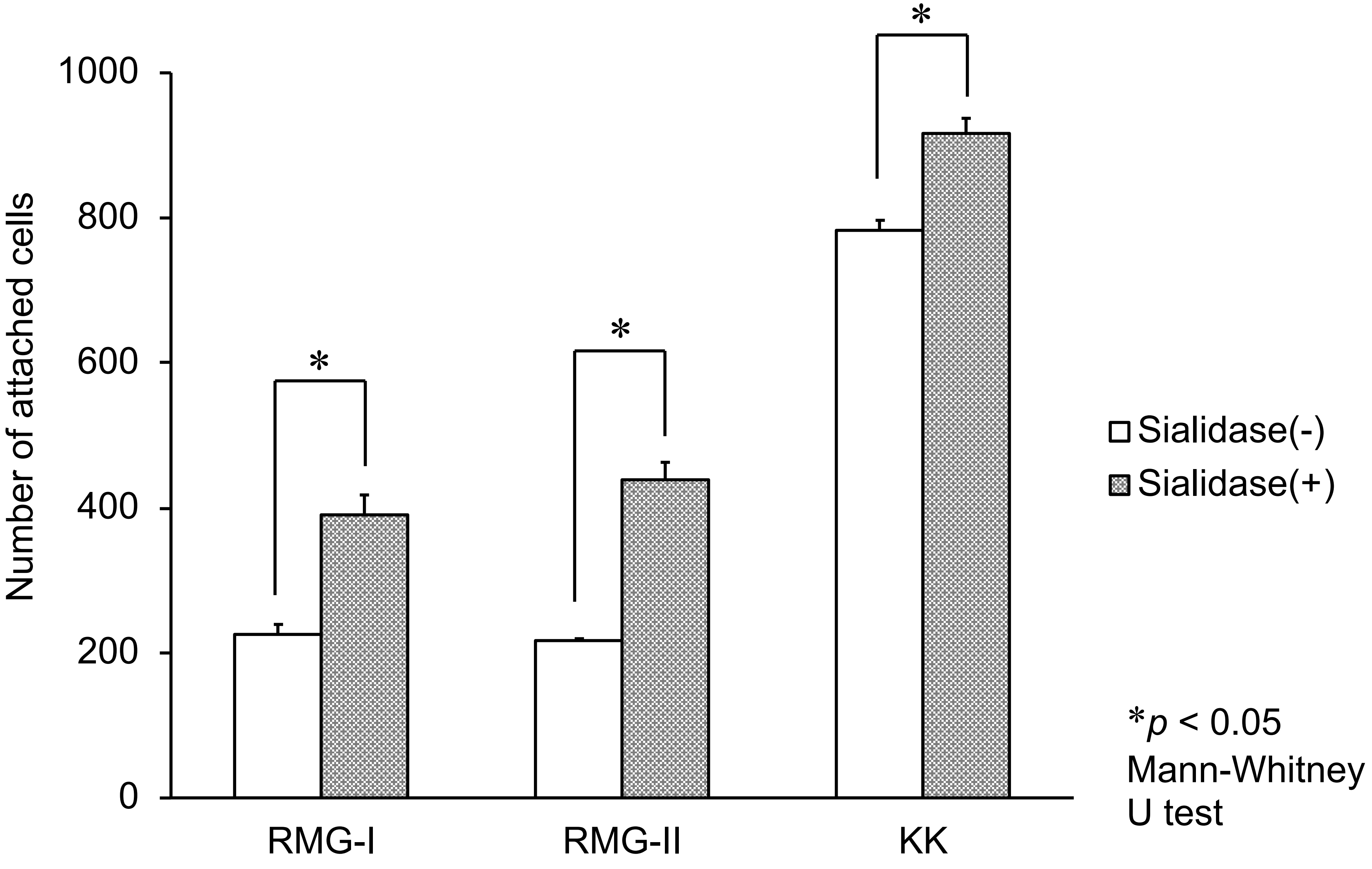

Surface expression level of sialyl-T MUC1 on three ovarian cancer cell lines as measured by flow cytometry using monoclonal antibody (mAb) MY.1E12.

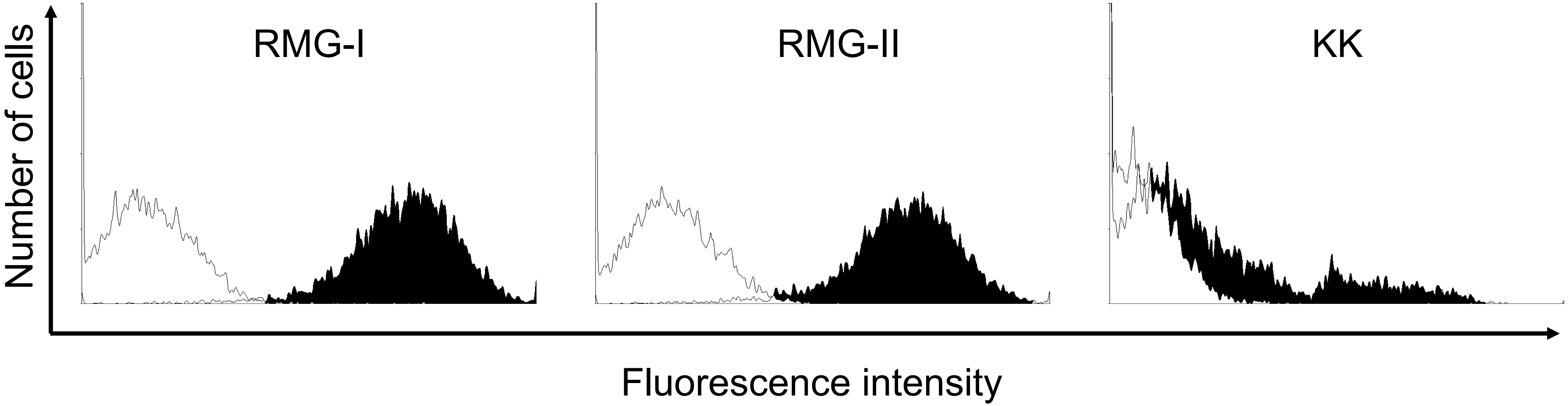

White shading: negative control (secondary antibody only)
Black shading: mAb MY.1E12

Supplementary Figure S3

Effect of sialidase treatment of peritoneal mesothelial cells on the adhesion of three ovarian cancer cell lines to mesothelial cells *in vitro*.

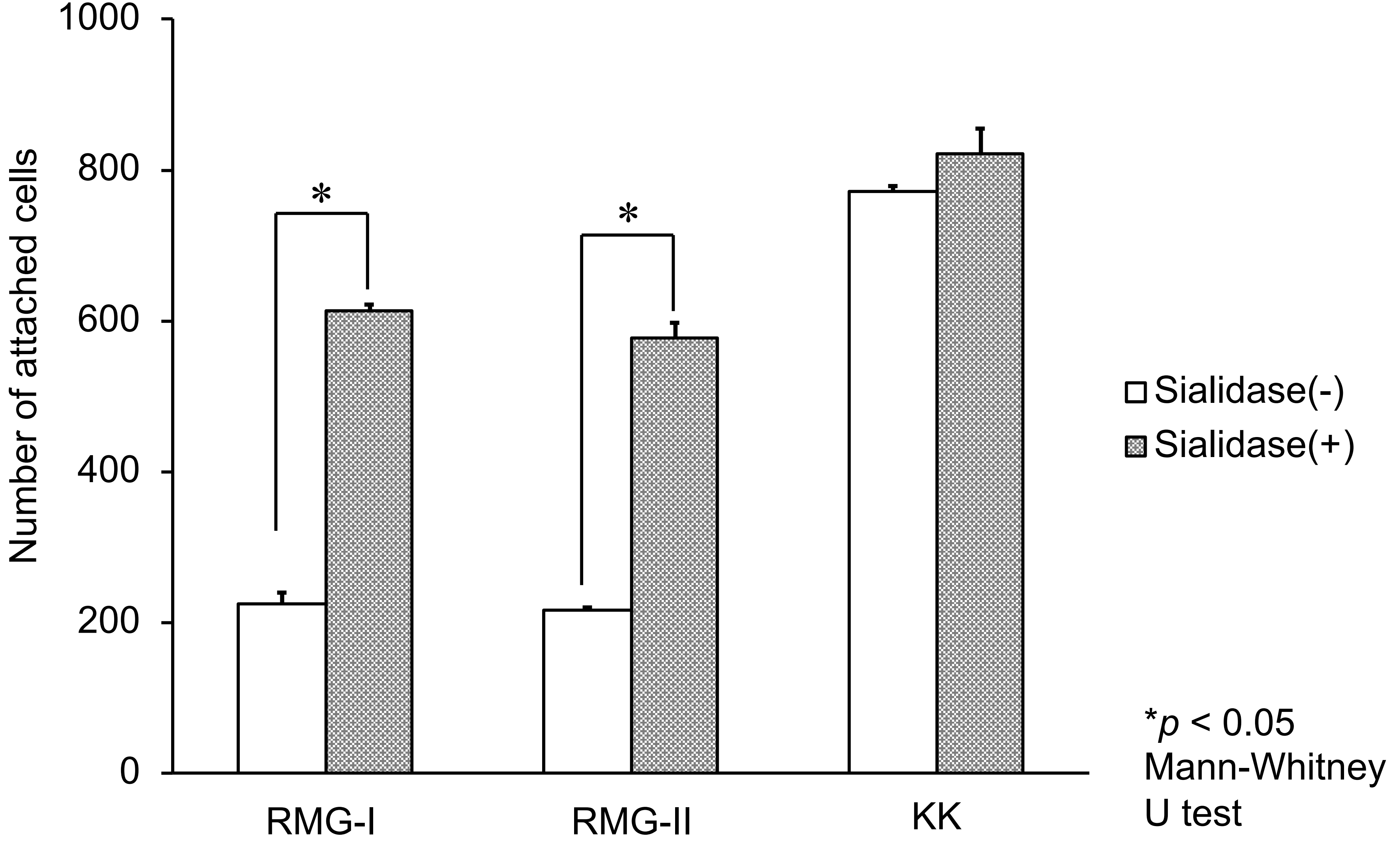

Supplement: Supplementary file 1 [file molecules-26-05962-s001.zip › molecules-1384497-supplementary.pdf]
